# Impact of drug legislation on crime reduction: a systematic literature review

**DOI:** 10.3389/fpubh.2026.1806036

**Published:** 2026-05-20

**Authors:** María Clara Torres-Rodríguez, Guisella Gonzabay-Medina, Elka Jennifer Almeida-Monge, Wilmer José Gavilánez-Rodríguez, Susana Lorena Toledo-Álvarez, Valeria Domenica Jara-Villavicencio

**Affiliations:** Universidad Estatal de Milagro, Milagro, Guayas, Ecuador

**Keywords:** crime, decriminalization, drug legislation, harm reduction, public policies

## Abstract

**Introduction:**

The impact of drug legislation on crime reduction remains a central issue in debates on public security and public health. Different regulatory models, ranging from punitive approaches to harm reduction and decriminalization, have produced heterogeneous outcomes depending on the legal and social context.

**Methods:**

A systematic literature review and bibliometric analysis were conducted following the PRISMA protocol. Studies published between 2000 and 2026 were identified through searches in Scopus and Web of Science. The analysis focused on publication trends, geographic distribution, and the effects of different regulatory models on crime-related outcomes.

**Results:**

The findings indicate that decriminalization and harm reduction policies are consistently associated with reductions in drug-related arrests, particularly among people who use drugs, without evidence of increases in violent or property crime in most analyzed contexts. In contrast, punitive and highly repressive approaches showed limited or counterproductive effects, including increased violence, recidivism, and persistent racial disparities in arrests. Academic production on this topic has increased substantially since 2018, with most studies concentrated in North America and Eastern Europe.

**Discussion:**

The effects of drug legislation vary according to the substance involved, the affected population, and the implementation context. The evidence suggests that public health-oriented and harm reduction strategies may be more effective than punitive approaches in reducing criminal justice harms. However, broader crime outcomes remain context-dependent, highlighting the need for comparative and context-sensitive research to inform future drug policies.

## 1 Introduction

The impact of drug legislation on crime reduction is a central topic in the debates on security policies and public health. Globally, countries have implemented different regulatory approaches ranging from strict criminalization to harm reduction and legalization models.

However, the effectiveness of these strategies remains controversial, as their consequences vary depending on the legal, social, and economic context (1). Beyond its function as a regulatory instrument, drug legislation can also be understood as part of broader institutional arrangements that shape exposure to punishment, surveillance, and unequal access to care. From a critical public health perspective, punitive drug control regimes may operate as forms of structural and state-sanctioned violence insofar as they disproportionately affect marginalized populations, reinforce stigma, and deepen health inequities rather than resolving the underlying determinants of drug-related harm (2–4).

Empirical evidence suggests that punitive policies do not always lead to crime reduction.

Studies have shown that criminalization and police repression can generate adverse effects, such as an increase in the prison population without a significant reduction in drug-related crimes (5, 6). By contrast, strategies based on decriminalization and harm reduction have been associated with decreases in drug-related arrests without consistent increases in violent crime, as observed in the reforms implemented in Oregon and Washington (7). In parallel, abolitionist public health has emerged as a framework that questions the routine use of policing, criminalization, and incarceration to manage social and health problems. Rather than locating safety in punitive systems, this perspective emphasizes prevention, collective care, redistribution of resources, and interventions aimed at the structural determinants of harm (8, 9). Applied to drug policy, this lens invites a shift from punishment-centered governance toward health-centered and rights-based responses.

In this context, this study aimed to analyze the impact of drug legislation on crime reduction through a systematic literature review and bibliometric analysis. Following the PRISMA protocol, this study identifies research trends and evaluates the effectiveness of different regulatory models.

The central question guiding this study is: What is the impact of drug laws on crime reduction?

Additionally, this study seeks to answer secondary questions regarding the evolution of academic production in this area and the identification of regulations with the greatest impact on crime reduction. By situating this question within current debates in public health, the study also considers whether drug policy reforms merely modify legal outcomes or, more fundamentally, challenge the institutional dynamics through which punishment contributes to social exclusion and health disadvantage.

From a theoretical perspective, the impact of drug legislation on crime can be better understood through key criminological frameworks. Deterrence theory suggests that stricter sanctions may discourage criminal behavior by increasing the perceived cost of committing offenses.

However, empirical evidence often contradicts this, especially in the case of drug-related crimes. On the other hand, labeling theory posits that criminalizing drug users reinforces deviant identities and social exclusion, potentially leading to higher recidivism and undermining rehabilitation. These theories help explain how different regulatory approaches may produce distinct outcomes in crime-related indicators. These criminological perspectives can be further strengthened by a structural violence approach, which helps explain how legal and institutional arrangements systematically expose certain groups to harm by restricting access to health, protection, and social inclusion (3). In this sense, the effects of drug legislation extend beyond formal crime indicators: policing and carceral exposure have been associated with adverse health consequences and with neighborhood-level patterns of violence and poor health, underscoring the need to examine drug policy through the combined lenses of crime, health justice, and inequality (10, 11).

Through this analysis, this study aims to provide empirical evidence that contributes to understanding the effects of drug legislation and informs the design of public policies related to crime reduction. This research is framed within the growing academic interest in this topic, reflected in the increase in publications in recent years highlighting the need for a data-driven approach to evaluate best practices in drug regulation (12). Accordingly, this review not only examines whether particular regulatory models are associated with changes in crime-related outcomes, but also whether they align with a broader public health orientation capable of reducing criminal justice harms, mitigating state violence, and addressing health inequities among people who use drugs and other affected populations.

## 2 Methodology

### 2.1 Research design and search protocol

This study adopts a systematic literature review (SLR) and a bibliometric analysis, following the guidelines of the PRISMA (Preferred Reporting Items for Systematic Reviews and Meta-Analyses) protocol. The objective was to analyze the relationship between drug legislation and crime reduction by identifying trends, gaps, and future research opportunities.

For formulating the research question, the PICo model was used:

- P (Population): National and international legal systems regulating drug consumption and trafficking.
- I (Intervention/Interest): Drug legislation and its impact on crime rates.
- Co (Context): Drug-related crime in different legal frameworks and time periods.

The main research question guiding this study is: What is the impact of drug laws on crime reduction?

The following secondary research questions (Q) were established:

- Q1: What is the trend in the publication of studies on drug legislation and crime, and which countries have the highest concentration of studies?
- Q2: What types of regulations have shown the greatest impact on reducing drug-related crimes?

In this study, “crime” is defined as offenses directly or indirectly associated with drug use and trafficking. This includes both violent crimes (e.g., homicides, assaults) and non-violent crimes (e.g., possession, drug dealing, property crimes related to drug use).

### 2.2 Databases and search strategy

Scopus and Web of Science (WoS) were selected as the primary scientific databases for indexing literature.

The search equation for WoS was: TS = (“drug policy” OR “drug legislation” OR “drug law” OR “narcotics regulation”) AND TS = (“crime reduction” OR “criminal activity” OR “violent crime” OR “homicide rate” OR “public safety”), and for Scopus, it was defined as: TITLEABS-KEY (“drug policy” OR “drug legislation” OR “drug law” OR “narcotics regulation”) AND TITLE-ABS-KEY (“crime reduction” OR “criminal activity” OR “violent crime” OR “homicide rate” OR “public safety”).

### 2.3 Inclusion and exclusion criteria

To ensure the relevance and quality of the selected studies, the following criteria were established:

Three criteria were defined:

1. Peer-reviewed articles indexed in Scopus and WoS (excluding book chapters, conference proceedings, theses, and non-peer-reviewed documents).
2. Studies written in English or Spanish.
3. Publications from 2000 to 2026.

### 2.4 Study selection process (PRISMA model)

The article selection process followed the PRISMA framework and consisted of four phases:

1. Identification: Documents identified through the initial search.
2. Screening: Removal of duplicate records and elimination after title and abstract review (studies not related to any key term in the search equations).
3. Eligibility: Removal of records where full-text access was not possible and elimination after full-text evaluation (studies that did not focus on drug legislation or crime outcomes, or where full-text access was not available).
4. Inclusion: Full-text reading and quality assessment.

### 2.5 Quality assessment of included studies

The quality of the selected studies was assessed using the Mixed Methods Appraisal Tool (MMAT), version 2018, which is designed to evaluate the methodological quality of empirical studies, including qualitative, quantitative, and mixed-methods research. Each study was independently reviewed and scored based on the criteria relevant to its design. Studies that did not meet at least three of the five MMAT criteria were excluded from the final analysis. This process ensured the inclusion of methodologically robust and relevant research in the systematic review.

### 2.6 Thematic analysis and categorization of regulatory strategies

To synthesize findings across the included studies, a thematic analysis was conducted. Regulatory strategies described in the literature were categorized into nine types based on their definitions and intended mechanisms:

- Tactical adjustment of sellers: Behavioral adaptations by drug dealers to avoid detection or arrest, such as changing sales locations or patterns.
- Media-driven/punitive: Policies influenced by media narratives that associate drug use with violence, promoting harsh enforcement responses.
- Criminalization: Legal frameworks that impose criminal sanctions for drug possession or use.
- Decriminalization: Policies that eliminate or reduce criminal penalties for possession of small drug quantities, often replaced with administrative measures.
- Drug interdiction: Actions aimed at reducing drug supply through seizures, border enforcement, or raids.
- Legalization: Full state regulation and control over the production, distribution, and use of certain substances.
- Harm reduction: Public health approaches that aim to minimize the adverse
- Consequences of drug use without necessarily reducing consumption (e.g., syringe exchange, overdose prevention).
- Police repression: Intensified police actions or operations targeting users or sellers, often associated with high arrest rates.
- Public health regulation: Approaches that treat drug use as a health issue, emphasizing prevention, treatment access, and social reintegration.

This classification enabled the synthesis of findings across diverse policy environments and study designs.

## 3 Results

**Figure 1:**
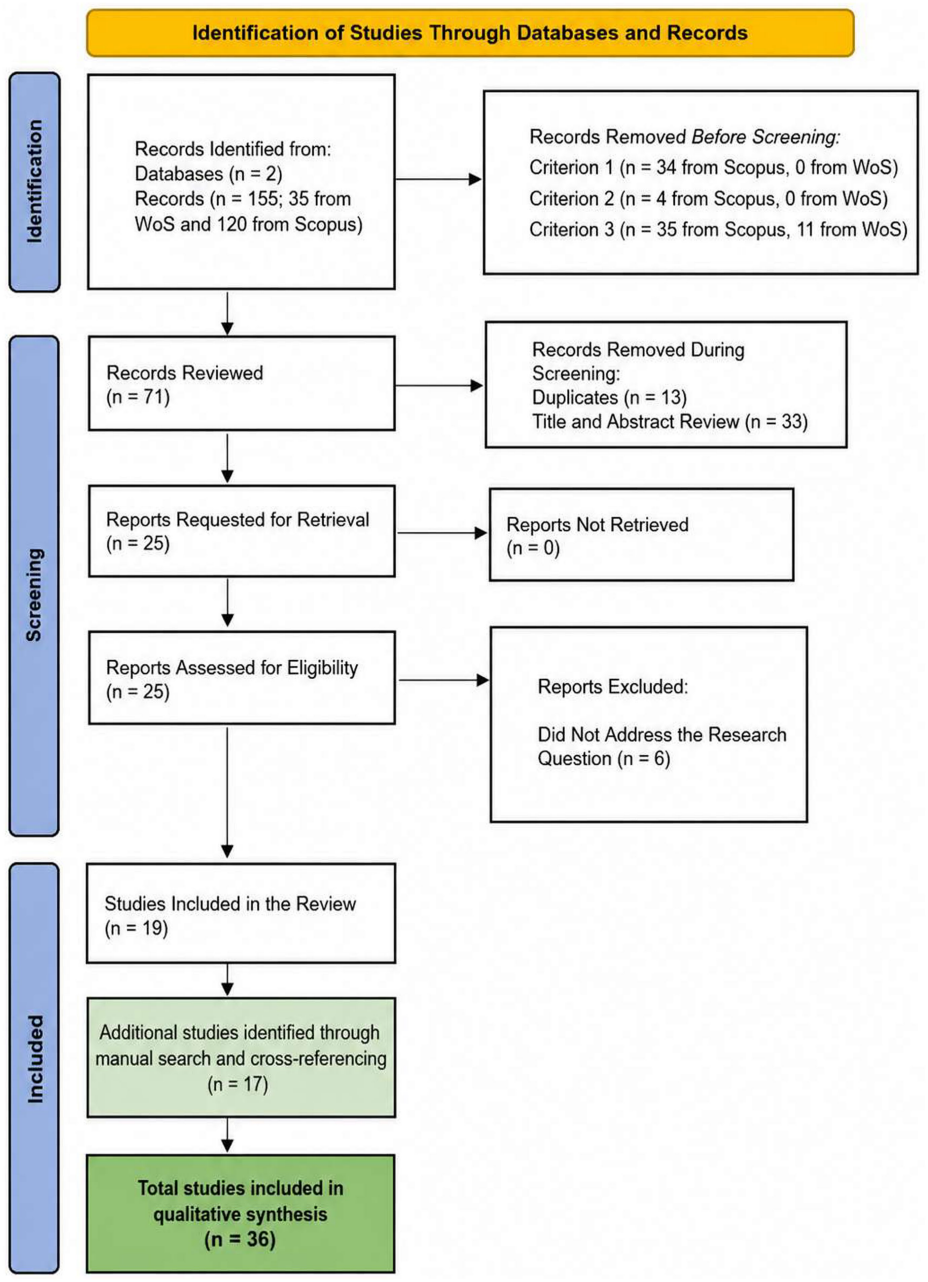
PRISMA Selection Process.

The initial search was conducted on February 25, 2025, yielding 155 records. During the identification process, 84 records were eliminated. In the screening phase, 46 documents were removed, and 6 articles were excluded as they did not address the research question (Figure 1).

Following the PRISMA framework, the initial database search in Scopus and Web of Science (2000–2026) identified 155 records. After screening and eligibility assessment, 19 studies met the inclusion criteria (Figure 1).

Subsequently, to address reviewer feedback regarding the limited number of studies and to ensure comprehensive coverage of the expanded time horizon (2000–2026), a complementary search was conducted. This included: (a) manual identification of key foundational studies published between 2000 and 2014 that were not captured in the initial search due to keyword specificity, and (b) cross-referencing from included studies and reviewer suggestions. This process yielded an additional 17 studies, resulting in a final corpus of 36 studies for analysis. The complete list, including studies from both the systematic database search and the complementary search, is presented in Table 1.

**Table 1:** List of studies reviewed.

Code	References	Country	Substance	Population	Outcome measured
01	Kuettel (38)	USA	General	General	Public attitudes toward drugs
02	Michaud et al. (16)	USA, Canada	General	Police, Users	Police-public health partnerships, trust in healthcare
03	Fromm and Blokland (33)	Brazil	Crack	Users	Displacement, urban conflict, mobility patterns
04	Rouhani et al. (20)	USA	General	General	De facto decriminalization, arrest trends, implementation barriers
05	Marotta et al. (52)	USA	Injection drugs	Police, Users	Police willingness to refer to harm reduction services
06	Davis et al. (7)	USA (OR, WA)	Possession	General	Arrests, violent crime rates
07	Soliman (53)	USA	General	General	Arrests, policing activity, offender behavior
08	Sohoni et al. (40)	USA	General	General	Media portrayals of drugs and violence
09	Kuryliuk et al. (5)	Ukraine	General	Incarcerated	Recidivism, effectiveness of penal system
10	Arredondo et al. (14)	Mexico	Possession	General	Arrests, police enforcement patterns
11	Babor et al. (1)	Global	General	General	Public health outcomes, policy effectiveness (review)
12	Pavlenko and Mozghova (27)	Ukraine	Psychoactive	Users	Psychological disorders, behavioral outcomes
13	Dolliver (25)	Poland	Multiple	Drug market	Drug interdiction effectiveness, seizure rates
14	Morales et al. (6)	Mexico	General	Users	Police crackdown effects, human rights violations
15	Fader (29)	USA	General	Sellers	Risk perception, behavioral adaptation to enforcement
16	Osborne and Fogel (32)	Canada	Cannabis	Recreational users	User perspectives on legalization, gateway effects
17	Gaines et al. (28)	Mexico	General	Users	Police enforcement patterns post-reform
18	Lunze et al. (34)	Russia	Injection drugs	Users	Stigma, human rights abuses, access to healthcare
19	Rad et al. (12)	Iran	General	General	Crime rates, judicial burden, legalization impacts
20	Agra (35)	Portugal	General	General	Decriminalization outcomes, treatment integration
21	Callaghan et al. (26)	Canada	Cannabis	General	Crime patterns post-legalization
22	Gaudreault et al. (13)	Canada (BC)	Illicit drugs	General	Drug-related offenses, decriminalization impact
23	Greer et al. (15)	Canada (BC)	General	Police, Users	Police discretion, implementation of depenalization
24	Gunadi and Shi (18)	USA	Cannabis	Racial minorities	Racial disparities in arrests
25	Kammersgaard (30)	Norway	General	General	Decriminalization proposals, tensions between punishment and help
26	Laursen and Jepsen (37)	Denmark	General	General	Mixed strategies, repression vs. welfare balance
27	Maier et al. (39)	USA	Cannabis	General	Public opinion, policy preferences
28	Martinez et al. (22)	USA (CA)	Injection drugs	PWID	Arrest rates, syringe exchange legalization
29	Plunk et al. (17)	USA	Cannabis	Youth, Adults	Arrests for cannabis possession
30	Resignato (23)	USA	General	General	Violent crime, drug enforcement effects
31	Rouhani et al. (19)	USA (Baltimore)	General	Racial minorities	Racial disparities in arrests post-decriminalization
32	Rouhani et al. (31)	USA (Baltimore)	General	Police, Users	Implementation barriers, police resistance
33	Shirley-Beavan (24)	Colombia	Basuco	Users	Direct violence, punitive policy effects
34	Single et al. (36)	Australia, USA	Cannabis	General	Decriminalization effects, enforcement practices
35	Stevens et al. (41)	Multiple	General	General	Alternatives to criminalization, realist review
36	Tran et al. (21)	USA (Philadelphia)	Cannabis	General	Arrests, racial disparities

The 36 selected articles for the literature analysis are summarized in Table 1.

**Figure 2:**
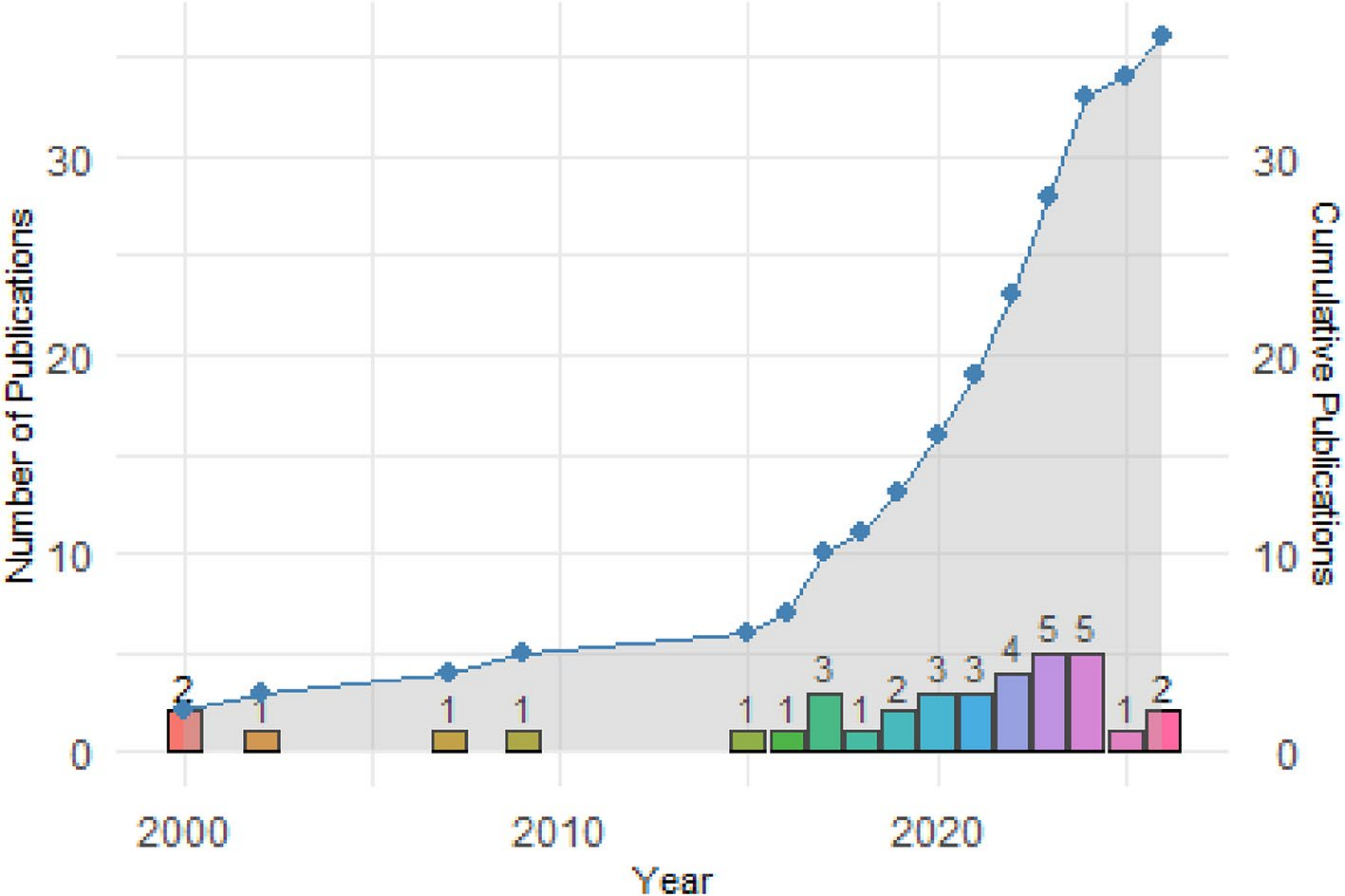
Annual and cumulative publications on drug legislation and crime (2000–2026).

Figure 2 shows the temporal distribution of the 36 studies included in this systematic review, sorted by year of publication between 2000 and 2026. The bars represent the annual number of studies included. As can be seen, the volume of research remained relatively low in the early 2000s, although there was a notable increase starting in 2017, coinciding with growing academic interest in drug policy reforms in North America and Europe. The upward trend becomes more pronounced starting in 2020, reaching its peak in 2024 with four publications. This growth reflects the increasing relevance of the topic and the expansion of empirical research evaluating different regulatory models. The cumulative line shows exponential growth, especially after 2018, indicating sustained academic attention to the relationship between drug legislation and crime.

### 3.1 Impact on arrests and police activity

Across the studies analyzed, the most consistent finding relates to drug-related arrests and police activity, rather than overall crime outcomes. However, the effects of drug policy reforms on arrests vary considerably depending on the substance, the population, and the context of implementation.

Studies evaluating decriminalization typically report significant reductions in arrests for drug possession among people who use drugs (PWUD). In Oregon and Washington, Davis et al. (7) documented substantial declines in arrests following decriminalization, without corresponding increases in violent crime. Similarly, Gaudreault et al. (13) observed that British Columbia’s decriminalization policy led to reductions in drug-related crimes during its first year of implementation.

However, these effects are not uniform across all jurisdictions. In Tijuana, Mexico, Arredondo et al. (14) found that the 2009 decriminalization reform had no significant impact on arrests for drug possession, and noted that police enforcement against drug users actually increased following the reform. This contrast highlights the critical role that local implementation and policing practices play in determining policy outcomes. Among studies focusing on the police, the results suggest that law enforcement practices often persist despite legal reforms. Greer et al. (15) and Michaud et al. (16) show that the police may continue to use discretionary practices that maintain criminalization in practice.

When breaking down the data by substance, the decriminalization and legalization of cannabis have had a clearly positive effect on reducing arrests. Plunk et al. (17) documented, following policy changes in the United States, a sharp decline in arrests for cannabis possession among youth and adults. However, Gunadi and Shi (18) as well as Rouhani et al. (19) highlighted the persistence of racial inequalities, as Black people continue to be arrested at a disproportionate rate even after reform, suggesting that formal policy changes do not automatically eliminate biased law enforcement practices.

Beyond formal decriminalization, de facto reforms have also been documented in which police, at their discretion, reduce criminal prosecution without legislative changes. Rouhani et al. (20) characterized new models of de facto decriminalization in the United States and noted that, while these approaches may reduce the number of arrests, they often face obstacles to implementation, including inconsistent police practices and public misinformation. Similarly, Tran et al. (21) analyzed cannabis-related criminal prosecution in Philadelphia and observed that, despite informal shifts in policing priorities, racial disparities persisted.

With regard to harm reduction interventions, Martinez et al. (22) demonstrated that the legalization of needle exchange programs in California significantly reduced arrest rates among people who inject drugs (PWID), suggesting that the decriminalization of secondary behaviors may have protective effects for vulnerable populations.

### 3.2 Effects on violent and property crime

The relationship between drug policies and violent crime is complex, often mediated by market dynamics and enforcement strategies. Studies from regions with high levels of drug-related violence, particularly in Latin America, demonstrate the inadequacy of punitive approaches.

For example, Resignato (23) conducted an early empirical analysis that demonstrated drug enforcement itself may be a more significant factor in violent crime than drug use. This suggests aggressive policing can paradoxically increase violence.

More recently, Shirley-Beavan (24) introduced the concept of “direct violence as drug-related harm” in her study of basuco (cocaine base paste) users in Bogotá, Colombia. Her analysis shows how punitive policies expose marginalized users to violence from state actors and criminal groups alike, and she argues that harm reduction approaches are essential to mitigating these harms. Together, these studies imply that policies focused solely on reducing supply and enforcing laws may exacerbate violence rather than reduce it.

The relationship between drug policy and crime also extends to drug supply interventions. Dolliver (25) analyzed drug interdiction in Poland over a 15-year period and found that seizure rates did not consistently correlate with reductions in trafficking offenses. This suggests that there are limitations to supply-side enforcement strategies. Similarly, Callaghan et al. (26) examined crime patterns following cannabis legalization in Canada and reported no significant increases in violent or property crime. This finding reinforces the argument that regulatory approaches need not compromise public safety.

### 3.3 Recidivism and behavior adaptation

Data on recidivism and behavioral adaptation suggest that punitive approaches may have unintended consequences. Studies focusing on incarcerated populations or those who have been in prison indicate that criminalization may increase recidivism rather than reduce crime. Kuryliuk et al. (5) found that Ukraine’s punitive drug policies failed to reduce reoffending rates, highlighting the limitations of carceral approaches.

The consequences of punitive approaches extend beyond recidivism and affect mental health. Pavlenko and Mozghova (27) highlighted that Ukraine’s drug policy, centered on criminalization, fails to address psychological disorders associated with psychoactive substance use, and recommended a shift toward harm reduction and the integration of mental health care. Similarly, Gaines et al. (28) documented that in Tijuana, despite decriminalization reforms, police enforcement against drug users increased, leading to higher rates of criminal charges and continued marginalization.

From the perspective of actors in the drug market, Fader (29) conducted in-depth interviews with drug sellers and found that the certainty of being caught was a greater deterrent than the severity of the punishment. Notably, sellers adapted their tactics to avoid arrest rather than exiting the trade entirely. This phenomenon is described as “selling smarter, not harder.” This tactical adjustment suggests that punitive policies may merely displace rather than eliminate drug market activity.

Qualitative studies of police behavior further illuminate the gap between policy intent and frontline practice. For example, Greer et al. (15) examined depenalization in British Columbia and found that police officers continued to use simple possession charges to manage marginalized populations, which undermined the stated goals of reform. Michaud et al. (16) examined police-public health partnerships and identified structural barriers that increase distrust of and avoidance of healthcare systems among drug users. This can limit the positive impact on crime reduction.

Kammersgaard (30) analyzed Norway’s decriminalization reform proposal, identifying tensions between punishment and assistance. He noted that proposals often maintain coercive elements under a rehabilitative facade. Similarly, Rouhani et al. (31) examined Baltimore’s de facto decriminalization and found that implementation barriers, including police resistance and public misinformation, constrained the policy’s impact. These findings underscore that formal policy changes do not automatically translate into transformative practices.

Collectively, these findings suggest that punitive approaches may displace criminal activity rather than eliminate it, while also generating unintended consequences such as increased recidivism, mental health harms, and the persistence of discretionary enforcement practices.

### 3.4 Differences by substance type

The effects of drug policies vary considerably depending on the substance in question.

Studies focusing on cannabis show relatively consistent results, including a reduction in arrests and a limited impact on violent crime (17, 32).

In contrast, studies addressing injectable drugs, crack, or synthetic opioids reveal more complex dynamics, often linked to marginalization, health risks, and structural violence (33, 34).

These findings suggest that the effects of policies are not independent of the substance, and conclusions must be interpreted accordingly.

### 3.5 Cross-jurisdictional variation and contextual factors

The meaning and implementation of drug policy reforms vary considerably from one jurisdiction to another. Consequently, similar policy labels (e.g., “decriminalization”) can lead to different outcomes depending on institutional capacity, law enforcement practices, and social context.

A clear example of this variation is Portugal’s decriminalization experience, documented by Agra (35). Often cited as a successful model, its results reflect a comprehensive approach that combined decriminalization with expanded treatment services and social reintegration programs. In contrast, Single et al. (36) found that cannabis decriminalization in Australia and the United States produced varying effects depending on local enforcement practices and cultural attitudes.

Denmark’s approach, characterized by Laursen and Jepsen (37) as an “ambivalent balance between repression and welfare,” illustrates the complexities of maintaining mixed strategies. The Danish case shows that even countries with strong welfare systems struggle to balance public-health goals with law enforcement priorities.

Public attitudes toward drug policy also play a role in shaping reform trajectories. Kuettel (38) found that public sentiment in the US has become more liberal toward drugs since the late 2000s, with punitive measures acting as a major negative predictor of public support. Similarly, Maier et al. (39) documented shifts in public opinion in favor of cannabis legalization, suggesting that changing social norms may create space for policy innovation. By contrast, media narratives often reinforce punitive responses; Sohoni et al. (40) showed that drug depictions in crime television exaggerate the link between substance use and violence, legitimizing severe enforcement.

Stevens et al. (41) conducted a realistic review of alternatives to criminalization for drug possession and developed program theory that emphasizes that outcomes depend on the interplay between mechanisms (e.g., stigma reduction, resource diversion), context (e.g., police culture, availability of treatment), and outcomes. That framework helps explain why Oregon’s decriminalization led to a visible decline in arrests, while similar reforms in Tijuana did not, and highlights the importance of contextual factors such as institutional capacity, police training, and the availability of alternative treatments.

The geographical distribution of the studies reflects this complexity. While North America produces the most research, studies from Europe, Latin America, and other regions provide important insights into how different institutional and cultural contexts shape policy outcomes. The limited number of studies from Africa, Asia, and the Middle East, excluding Iran (12), represents a major gap in understanding the global applicability of different regulatory models.

**Figure 3:**
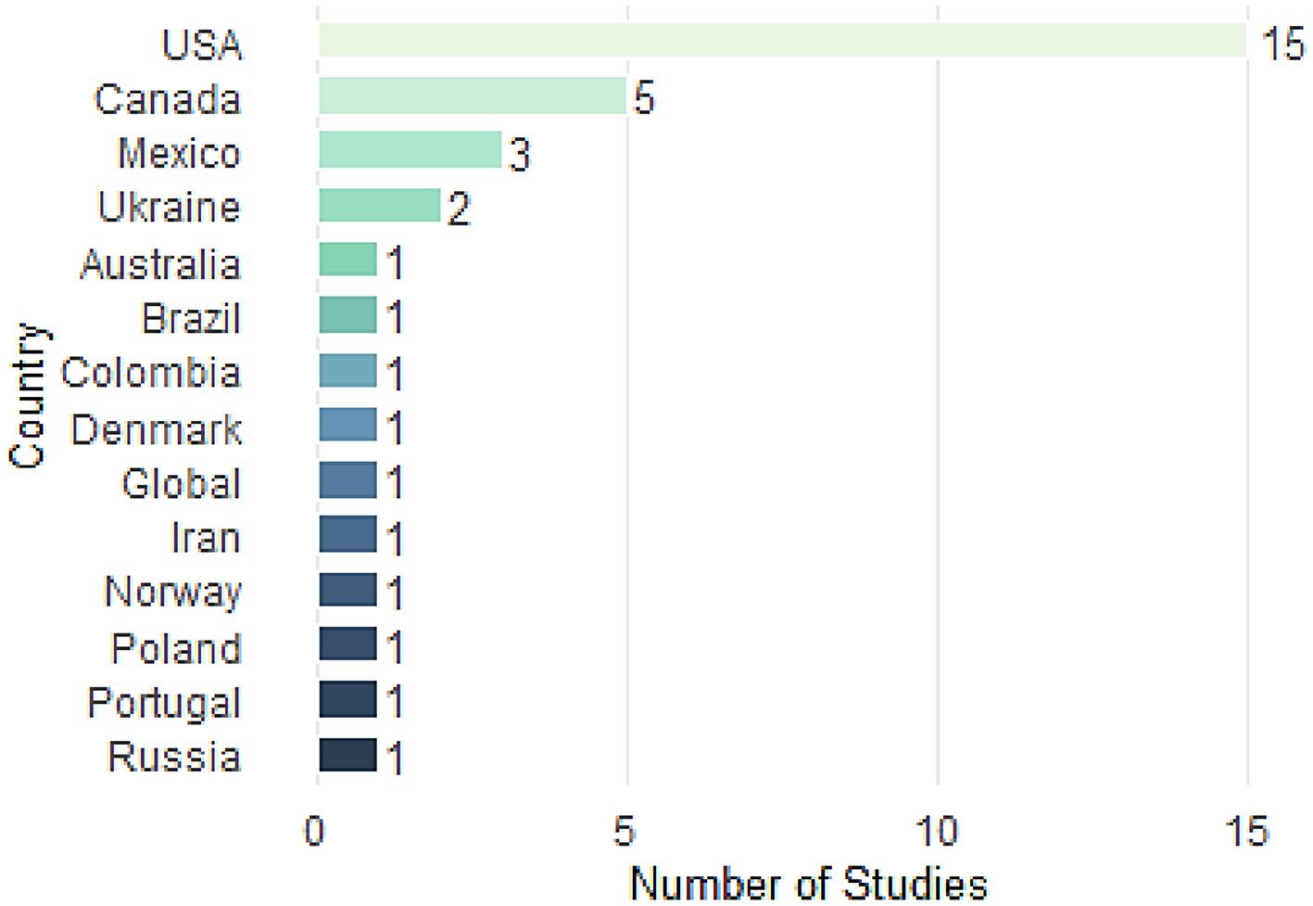
Number of studies per country based on corresponding author affiliation.

Figure 3 shows the geographic distribution of the 36 included studies according to the corresponding author’s affiliation. The United States accounts for the largest number of studies (*n* = 15), reflecting its long history of punitive drug policies, recent decriminalization and legalization reforms at the state level, and the availability of longitudinal data for empirical analysis. Canada (*n* = 5) and Mexico (*n* = 3) also contribute substantially, likely due to their respective drug policy reforms: the legalization of cannabis in Canada in 2018 and Mexico’s decriminalization reform in 2009. Studies from Europe are represented by Ukraine (*n* = 2), Denmark, Norway, Poland, Portugal, and Russia (*n* = 1 each), while other regions such as South America (Brazil, Colombia), the Middle East (Iran), and Australia have limited representation. The concentration of research in North America and Eastern Europe highlights a significant geographical gap in the literature that met the inclusion criteria, particularly in Africa, Asia (excluding Iran), and most of South America, underscoring the need for further studies in these regions to assess the applicability of different regulatory models across various institutional and cultural contexts.

## 4 Discussion

The findings of this study reveal that drug policies have a significant impact on crime-related outcomes, but their effects are not uniform. As shown in the results section, outcomes vary considerably depending on the population studied (e.g., general population, people who use drugs, sellers), the type of substance (e.g., cannabis vs. opioids), the jurisdictional context, and the quality of implementation. This heterogeneity underscores the need to move beyond generalized claims about policy effectiveness. From the perspective of abolitionist public health, this heterogeneity also suggests that drug legislation should not be interpreted only in terms of legal design or crime-control efficiency, but also in terms of the harms produced by the institutions through which it is enforced. In this sense, punitive drug policy may operate as a form of state-sanctioned and structurally mediated harm when it disproportionately exposes marginalized populations to surveillance, coercion, incarceration, and reduced access to care, thereby reinforcing existing health inequities (4, 42).

First, an important distinction must be made between different operationalizations of “crime.” The evidence consistently shows that decriminalization is associated with reductions in drug possession arrests, particularly among people who use drugs (PWUD). However, these reductions should not be interpreted as equivalent to decreases in violent or property crime, which are measured at the population level and are influenced by different mechanisms. Across the reviewed studies, the relationship between drug policy reforms and violent crime remains mixed and context-dependent, with several studies reporting null effects. This distinction is analytically important because, even where broader crime indicators remain unchanged, reductions in arrests may still represent a meaningful decline in criminal justice harms. Research on drug-use criminalization has shown that punitive legal environments are associated with adverse health consequences, including elevated HIV-related risks and barriers to prevention and treatment among people who inject drugs, which means that a reduction in arrest exposure may itself be a relevant public health outcome (43, 44).

Second, the effects of drug policies vary substantially across populations. Studies focusing on PWUD consistently report reductions in arrests and, in some cases, improved access to health services under decriminalization and harm reduction frameworks. In contrast, studies examining general population crime rates do not show consistent reductions in violence. Similarly, research on incarcerated populations suggests that punitive approaches may increase recidivism, while studies on policing practices highlight the persistence of discretionary enforcement even after formal legal reforms. These differences underscore that policy effects are not uniform and cannot be generalized across groups. Seen through a health equity lens, these population differences are not secondary findings but evidence that punitive drug governance is unevenly distributed across social groups. Scholarship on mass incarceration has shown that criminal justice exposure is linked to unequal burdens on well-being and health, especially among racially marginalized and socioeconomically disadvantaged populations, making it necessary to interpret drug policy effects not only by aggregate crime outcomes but also by their differential consequences across groups (45, 46).

Third, the results differ depending on the type of substance involved. Cannabis-related reforms show relatively consistent outcomes, including reductions in arrests and limited impact on violent crime. However, policies targeting substances such as opioids, crack, or injection drugs are associated with more complex dynamics, often linked to structural vulnerability, health risks, and marginalization. This indicates that drug policy effects are not substance-neutral and should be interpreted accordingly. This variation by substance supports a more differentiated interpretation of reform. In contexts involving injecting drug use and other highly stigmatized forms of consumption, harm reduction has been shown to provide not only clinical benefit but also ethical and public health value by reducing avoidable harms, supporting autonomy, and improving engagement with care. Likewise, supervised consumption interventions have been associated with positive public health and public order outcomes, suggesting that alternatives to punishment may be particularly relevant in settings where criminalization intersects with acute health risk (47, 48).

Fourth, cross-jurisdictional variation plays a critical role. Policies labeled as “decriminalization” or “harm reduction” differ significantly in their legal thresholds, enforcement practices, and accompanying health infrastructures. For example, Portugal’s model combines decriminalization with extensive treatment systems, whereas in other contexts, such as Tijuana or Baltimore, implementation is partial or inconsistent. These differences help explain why similar policies produce divergent outcomes. This point is especially relevant for the editor’s concern with abolitionist approaches because it shows that legal reform alone does not necessarily alter the underlying organization of punishment. Policy frameworks may adopt the language of harm reduction while remaining weak in substance, implementation, or institutional support. Comparative policy analysis has shown that jurisdictions can rhetorically endorse harm reduction without clearly defining interventions, reducing stigma, or recognizing people who use drugs as legitimate participants in policy design, thereby limiting the transformative reach of reform (49).

The findings can be further understood through criminological theory. Labeling theory helps explain why criminalization may increase recidivism and social exclusion, as individuals labeled as offenders face barriers to reintegration. Deterrence theory, particularly its emphasis on the certainty rather than severity of punishment, provides insight into why punitive approaches often fail to eliminate drug-related activity, as individuals and markets adapt to enforcement strategies rather than cease operations. Together, these frameworks suggest that the limited effectiveness of punitive policies is not anomalous but consistent with established theoretical expectations. These interpretations can be deepened by public health scholarship that treats incarceration not merely as a legal consequence, but as a mechanism that structures morbidity, exclusion, and inequality. In this sense, punishment is not only a response to drug-related conduct; it can itself become a generator of health disadvantage. This argument is reinforced by work showing that mass incarceration widens health inequality and that reconstructive justice requires moving beyond carceral responses toward policies centered on public health and social repair (42, 50).

Importantly, the review identifies implementation as a central mediating factor. Several studies show that legal reforms do not automatically translate into changes in practice. Police discretion, institutional capacity, and public awareness can significantly influence outcomes, sometimes maintaining patterns of criminalization despite formal policy changes. From this standpoint, implementation is not simply a technical matter but a determinant of whether reform reduces or reproduces institutional harm. Harm reduction scholarship has emphasized that effective responses require explicit principles such as humanism, pragmatism, autonomy, and accountability in healthcare and service systems. When those principles are absent, punitive logics can remain embedded in frontline practice even after formal policy shifts, weakening the public health potential of reform (43, 51).

Overall, the evidence does not support a uniform conclusion regarding the effectiveness of any single regulatory model in reducing all forms of crime. Instead, it suggests that drug policies have differentiated effects depending on the outcome measured, the population affected, the substance involved, and the context of implementation. Taken together, these findings support a more critical interpretation of drug policy. Decriminalization and harm reduction appear more promising than punitive approaches in reducing criminal justice harms, but their broader significance lies in whether they also lessen the institutional production of vulnerability, stigma, and unequal health risk. From an abolitionist public health perspective, the relevant question is therefore not only whether a policy reduces arrests or selected crime indicators, but whether it contributes to displacing carceral responses with structures of care, equity, and collective safety (4, 42).

### 4.1 Directions for future research

Considering the geographic concentration of current literature, future research should explore the comparative effectiveness of drug legislation across different socioeconomic contexts. Specifically, studies comparing the impact of similar regulatory models in low-income and high-income countries could provide critical insights into how local economic conditions, institutional capacity, and public infrastructure mediate outcomes. Such comparative analyses would help bridge the current knowledge gap and support the global development of more context-sensitive drug policies. Additionally, future research should disaggregate outcomes by substance type, population, and crime category to avoid conflating distinct phenomena under the umbrella term “crime reduction.”

### 4.2 Study limitations

One of the main limitations of this study was the selection of information sources. Only publications indexed in the Web of Science (WoS) and Scopus were considered, which may exclude relevant literature available in other databases, government reports, or studies not indexed on these platforms. This could introduce biases in the representation of current knowledge of the relationship between drug legislation and crime.

Another important limitation lies in the inherent biases of the bibliometric analysis. Because the search was restricted to Scopus and Web of Science, studies published in regional or non-indexed journals may have been excluded, particularly those from low- and middle-income countries. Furthermore, the focus on English and Spanish publications introduces language bias, potentially omitting relevant studies published in other languages, such as Russian, Mandarin, or Arabic. These constraints may skew the representation of global research efforts and limit the generalizability of our findings. Future research should consider expanding the language and database scope or triangulating bibliometric data with gray literature and local sources to improve comprehensiveness.

Another limitation was the lack of comparable data among the analyzed studies. Because the reviewed research employs heterogeneous methodologies, a direct comparison of the results is challenging. Some methodological differences include the definitions of crime used, period of analysis, and control variables used in empirical studies. This diversity of approaches may limit our ability to draw generalizable conclusions.

Finally, the impact of drug laws on crime cannot be analyzed in isolation, as it is influenced by multiple external factors. Security policies, socioeconomic conditions, drug trafficking dynamics, and changes in the public perception of drug use can affect the observed outcomes in different countries. The interaction of these elements can lead to variations in the effectiveness of regulations, underscoring the need for multidimensional analyses in future studies.

## 5 Conclusion

This systematic review demonstrates that the relationship between drug legislation and crime is complex and cannot be reduced to a single generalized effect. The findings show that decriminalization and harm reduction policies are consistently associated with reductions in drug-related arrests, particularly among people who use drugs. However, these effects should not be interpreted as reductions in overall crime, as evidence on violent and property crime remains mixed and context-dependent.

The review also highlights that policy outcomes vary significantly across populations, substances, and jurisdictions. Cannabis-related reforms tend to produce more consistent results, while policies addressing other substances are linked to more complex social and health dynamics. Additionally, differences in implementation, institutional capacity, and enforcement practices play a decisive role in shaping outcomes, limiting the comparability of results across contexts.

Rather than identifying a universally effective model, the evidence suggests that drug policy impacts are conditional and mediated by multiple interacting factors. These include the definition of outcomes, the characteristics of the affected population, and the broader social and institutional environment.

From a policy perspective, the findings indicate that reforms oriented toward public health and harm reduction can reduce the criminal justice burden associated with drug use, particularly in terms of arrests. However, expectations regarding broader crime reduction should remain cautious, as these outcomes depend on mechanisms that extend beyond drug legislation alone.

Future research should prioritize comparative and context-sensitive analyses, with clearer differentiation of crime indicators, populations, and substances, to improve the precision and applicability of findings in this field.

## Data Availability

The original contributions presented in the study are included in the article/supplementary material, further inquiries can be directed to the corresponding author/s.
